# Characterization of exosomal microRNAs in preterm infants fed with breast milk and infant formula

**DOI:** 10.3389/fnut.2024.1339919

**Published:** 2024-01-18

**Authors:** Eun-Bit Kim, Jun Hwan Song, Linh Nguy-Hoang Le, Ho Kim, Ji Won Koh, Yekyeng Seo, Hwal Rim Jeong, Hyun-Taek Kim, Seongho Ryu

**Affiliations:** ^1^Soonchunhyang Institute of Med-bio Science (SIMS), Soonchunhyang University, Cheonan, Republic of Korea; ^2^Soonchunhyang University Cheonan Hospital, College of Medicine, Soon-chunhyang University, Cheonan, Republic of Korea; ^3^Department of Integrated Biomedical Science, Soonchunhyang University, Cheonan, Republic of Korea; ^4^Department of Pediatrics, College of Medicine, Soonchunhyang University, Cheonan, Republic of Korea

**Keywords:** preterm infant, exosomes, miRNA, small RNA sequencing, breastfeeding, serum, urine

## Abstract

Breastfeeding not only reduces infection-related morbidity, but also increases growth of preterm infants. Advantages of breast milk (BM) for preterm infants are significant. They continue to be studied. However, because not all preterm infants can receive breastfeeding, bovine-based infant formula (IF) is used as an alternative, which may increase the risk of several preterm complications. Exosomes isolated from biofluids are emerging as biomarkers in research of various diseases. Here, we characterized miRNA contents of exosomes in urine and serum samples of preterm infants who were BM and IF fed and performed transcriptomic analysis of small RNA libraries. We identified significantly up-regulated 6 miRNAs and 10 miRNAs, respectively. Gene Ontology (GO) analysis revealed that target genes of these miRNAs might participate in neuronal development, immunity modulation, detoxification of reactive oxygen species, and transmembrane exchange. Our data suggest that exosome-based systemic screening for preterm infants with breastfeeding might be a screening tool for identifying target molecules involved in therapy for preterm infants in neonatal intensive care unit (NICU) and for future application as nutraceutical formulations or pharmaceuticals.

## 1 Introduction

Preterm infants who are born extremely early (before 37 completed weeks) commonly have several complicated health problems (1, 2). According to WHO data, about 15 million infants are born prematurely every year. This trend does not tend to decrease. Because preterm infants are born at a crucial period for brain and body development, more than 1 million preterm infants die in their first year or are treated in neonatal intensive care units (NICUs) (3). Most NICUs encourage parents to keep the baby with kangaroo care because this can stimulate breast milk (BM) expression and decrease the stress level of both mother and infant (4). Advantages of BM for preterm infants are significant. They continue to be studied (5, 6). Breastfeeding is an effective way to enhance health and development, particularly for preterm infants, although this is controversial (7, 8). BM includes non-nutrient factors containing oligosaccharides, lipids, antibodies, immunoglobulins and lactoferrin (9). Premature infants are at high risk for neurodevelopmental and respiratory impairments later in life (10, 11). BM consumption is positively associated with later cognitive outcomes in full-term cohorts (7, 12, 13). However, such association in preterm infants has not been clearly identified yet (14, 15). A recent study has suggested a positive effect of BM containing linoleic acid (LA) and α-linolenic acid (ALA) on brain development of preterm infants. These precursors of long-chain polyunsaturated fatty acids (PUFAs) ω3 and ω6 are critical for brain development of preterm infants (16). In addition, BM fed premature infants have significantly lower values of oxidative stress metabolites in urine compared formula fed groups (17). Despite these beneficial effects of BM, the precise role of BM on premature infants about systemic modulations remains unclear. Furthermore, mothers’ milk is sometimes unavailable or inadequate for a few reasons, leading to preterm infants being fed with artificial formula alternative (18). Infant formula (IF) is designed as a substitute for BM to satisfy nutritional requirements of infants. Despite IF containing a higher caloric density and protein than BM, some studies reported significantly lower prevalence of necrotizing enterocolitis in BM-fed group than in IF-fed group (19), especially in preterm infants (20). Moreover, BM feeding can reduce cases of comorbidities such as death, sepsis, bronchopulmonary dysplasia, and severe retinopathy of prematurity (ROP) in infants more than IF feeding (21–26).

Mammalian cells can secrete exosomes that are found in most types of human biofluids such as serum and urine (27). Exosomes are lipid bilayer nano-sized (50–200 nm) vesicles that can protect their cargos against degradation. They can also transport regulatory cargos including miRNAs to adjacent and distant cells. However, they cannot replicate (28). Exosomes can promote a particular phenotype. They are involved in cell-to-cell communication by transferring their cargos (29, 30). Analysis of exosomes in biofluid samples collected non-invasively could detect pathological disorder or alterations (31). Thus, it can be used to evaluate conditions of premature infants in NICU. MicroRNAs (miRNAs) are endogenous small molecules with lengths of 18–25 nucleotides. They are involved in the regulation of gene expression after transcription (32, 33). They play a critical role in a wide range of biological processes and serve as useful diagnostic biomarkers in various diseases (29, 30). Recent studies have demonstrated the theoretical role of serum miRNA-495-5p levels in premature infants associated with bronchopulmonary dysplasia (34). Some plasma miRNAs have diagnostic potential in preterm infants with respiratory distress syndrome (RDS) (35). Preliminary evidence has indicated that urine extra cellular vesicles derived miRNAs can be used as biomarkers for necrotizing enterocolitis in premature infants (36). Moreover, many studies have investigated the application of exosomal miRNAs as biomarkers or therapies in the treatment of preterm infants (37–40). Multiple studies have reported changes in compositions and miRNAs within the body through diet or dietary regulation. There are also attempts to apply these changes to treatments. Some studies have reported that miRNAs themselves can be modulated by feeding, suggesting that dietary manipulation might be a promising therapeutic strategy (41–43).

Numerous studies have compared BM feeding and IF feeding in premature infants (44–46). However, few studies have measured gene expression systemically modified by BM and IF feeding using exosomal miRNAs derived from serum or urine samples of preterm infants. The aim of this research was to gain a deeper molecular understanding of the influence of BM or IF intake by preterm infants. To achieve this aim, exosomal miRNA transcriptional profiling was performed for preterm infant urine and serum samples to compare between BM-fed and IF-fed groups. Our hypothesis was that miRNAs differentially expressed in urine and serum after BM consumption would be highly relevant to lower health risk and that they could be harnessed as potential therapeutics for treating related complications in preterm infants. We therefore tried to acquire transcriptional features of exosomes derived from preterm infants after breastfeeding for future applications in neutraceutical formulas or pharmaceutical substances for the care of preterm infants.

## 2 Results

**Figure 1:**
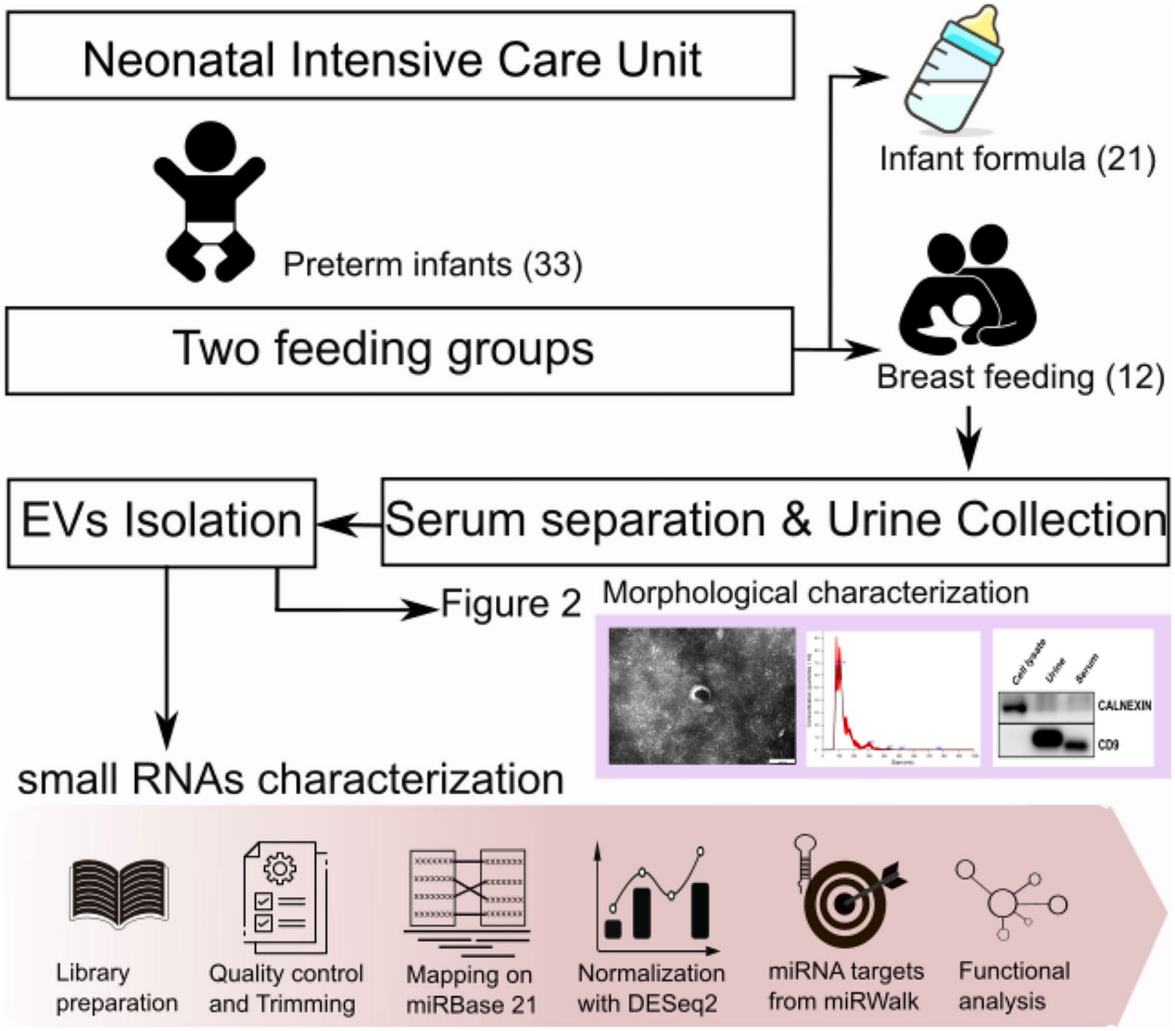
Experimental overview and bioinformatic pipeline (lower pink arrow) of this study.

A total of 33 preterm infants were recruited for this study. Complete overview of distinct experiments and analyses accomplished in this study is shown in Figure 1. The figure denotes sample collection process and morphological characterization of isolated exosomes with figuration of results. The lower pink arrow represents bioinformatic pipelines applied for small RNAs sequencing data analysis.

### 2.1 Characteristics of objectives

We recruited 33 premature infants in our study. Infants were classified with two groups: the BM group (*n* = 12) that was fed human milk and the IF group (*n* = 21) that was fed exclusively infant formula. The IF group consumed one commercial brand containing 67 kcal and 2.1 g/100 mL of proteins. There were no significant differences in gestational age, birth weight, height, head circumference, maternal age, diabetes, hypertensions, or body mass index between the two groups. Only multiple birth rate was significantly different (*p* < 0.001) between BM and IF groups. Demographic and clinical characteristics of premature infants are shown in Table 1. Urine and serum samples were collected before infants were discharged from NICU. Average age was 57 ± 25.24 days for the BM group and 55.76 ± 39.95 days for the IF group.

**Table 1:** Demographic and clinical characteristics of the study groups.

Characteristic	Breast-fed (*n* = 12)	Formula-fed (*n* = 21)	*p*-value
Gestational age (wk)	29 + 0.86 ± 3.39	30.65 ± 2.55	0.487
Birth weight (g)	1263.12 ± 433.7	1496.67 ± 552.62	0.1888
Birth height (cm)	38.25 ± 4.92	39.17 ± 3.89	0.5861
Birth head circumference (cm)	30.96 ± 3.61	32.24 ± 2.63	0.2963
Male	6 (50%)	12 (57.14%)	0.9736
Multiple birth	0 (0%)	15 (71.43%)	<0.001
SGA	4 (33.33%)	4 (19.05%)	0.6178
Apgar at 1 min	4.92 ± 2.57	5 ± 1.87	0.9228
Apgar at 5 min	7.08 ± 1.68	7.71 ± 1.85	0.3261
Maternal Age (yrs)	34.08 ± 3.45	36.29 ± 3.95	0.1067
Maternal diabetes*	5 (41.67%)	6 (28.57%)	0.7011
Maternal hypertension*	6 (50%)	13 (61.9%)	0.7645
Maternal body mass index (kg/m^2^)	29.22 ± 3.3	28.55 ± 2.68	0.5687
Sample collection date (d)	57 ± 25.24	55.76 ± 39.95	0.3994

Value are expressed as mean ± standard deviation or number (%).

SGA, small for gestational age.

*The case of both Maternal diabetes and Materanl hypertension (*n* = 3 in Breast-fed and *n* = 4 in Formula-fed).

### 2.2 Morphologic characterization of exosomes

**Figure 2:**
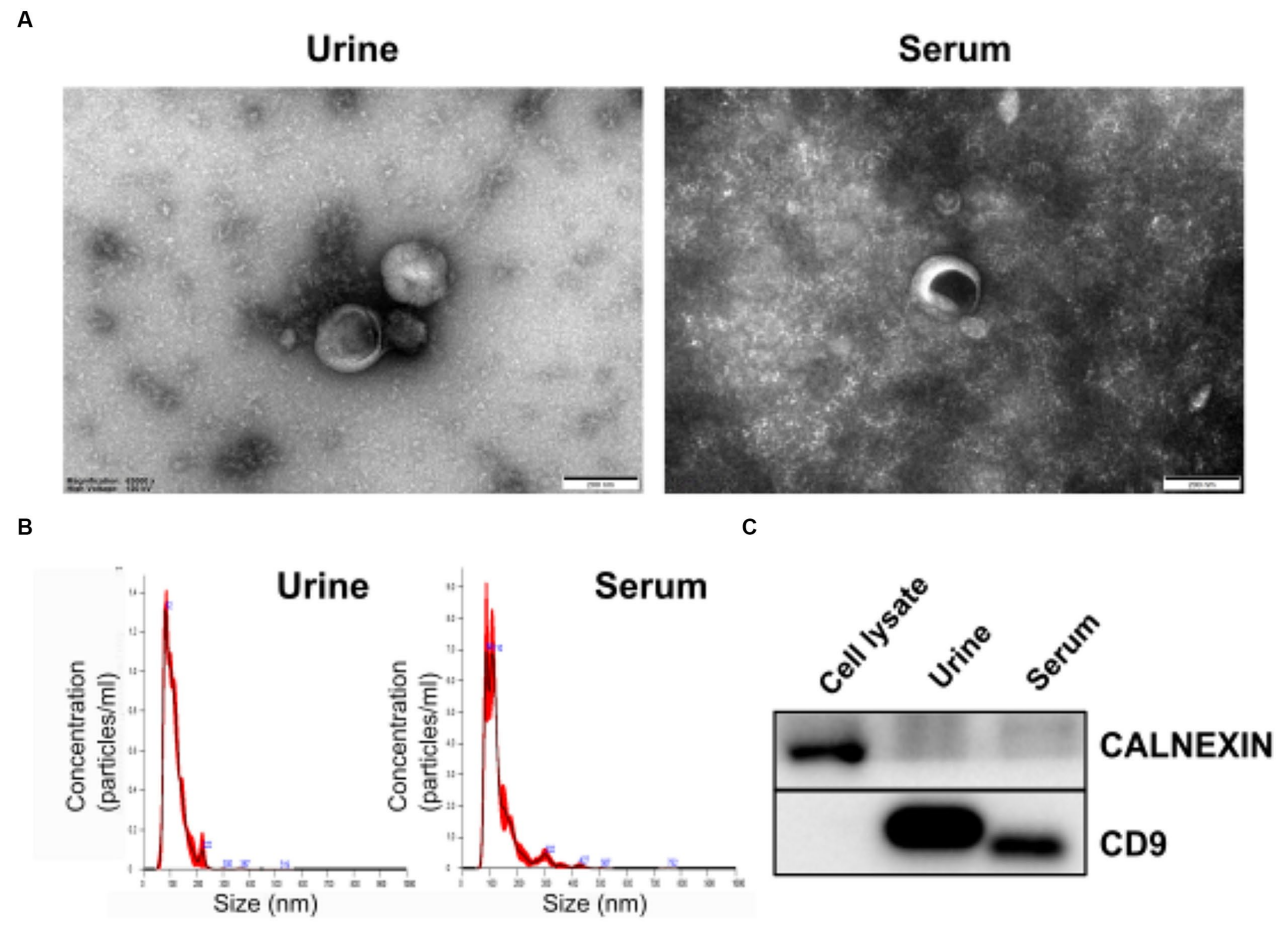
Morphological characterization of isolated exosomes. **(A)** Transmission electron microscopy (TEM) image revealing the morphology of exosomes and presence of vesicles with sizes under 200 nm. **(B)** Size distribution of exosomes measured with a Nanosight NS300 (NTA). The peak of the particle size was 82 nm for urine exosomes and 86 nm for serum exosomes. **(C)** Expression levels of exosomal membrane marker CD9 (Cluster of Differential 9) and endoplasmic reticulum marker CALNEXIN in isolated vesicles were determined by western blotting.

To identify exosomes in urine and serum samples of BM and IF groups, we purified exosomes and performed transmission electron microscopy (TEM). Purified exosome particles were spherical with diameters of less than 200 nm and a complete membrane structure (Figure 2A). Nanoparticle tracking analysis (NTA) revealed that diameters of exosomes isolated from urine samples of preterm infants had a mean size of 111.5 nm and a mode of 81.0 nm. Exosomes isolated from serum samples of preterm infants were characterized by a mean size of 134.5 nm and a mode of 85.6 nm. Concentrations of exosomes in urine and serum samples were approximately 7.9 × 10^11^ and 9.94 × 10^11^ particles/ml, respectively (Figure 2B). Immunoblotting showed that *CD9*, an exosome marker, was detected in both exosomes, but not in cell lysates (Figure 2C). An endoplasmic reticulum marker *CALNEXIN* was used to evaluate potential contamination by non-exosomal molecules. Compared with dark bands of cell lysates, only faint bands could be detected in both exosomes separated from urine and serum samples, indicating that there were a few contaminations of non-exosomal vesicles in isolated exosomes from biofluids.

### 2.3 Overview of small RNA sequencing

To characterize small RNA profiles of urine and serum exosomes, we prepared small RNA libraries from all premature infants in the test cohort. All reads were mapped to the human genome (hg38) and small RNA databases for miRNA (Supplementary Table S1). From 66 libraries, we obtained 2,982,090,421 raw reads from Illumina paired-end sequencing. After trimming and cleaning low-quality reads, 1,483,794,616 processed reads were generated. An average of 9,171,629 reads per library were mapped to the reference genome, with an average mapped rate of 39.6% (Supplementary Table S1). Through the alignment step, mappable cleansed reads were annotated to miRNA and a total of 2,656 miRNAs were detected in urine and serum exosomes.

### 2.4 Differentially expressed miRNAs in exosomes

**Figure 3:**
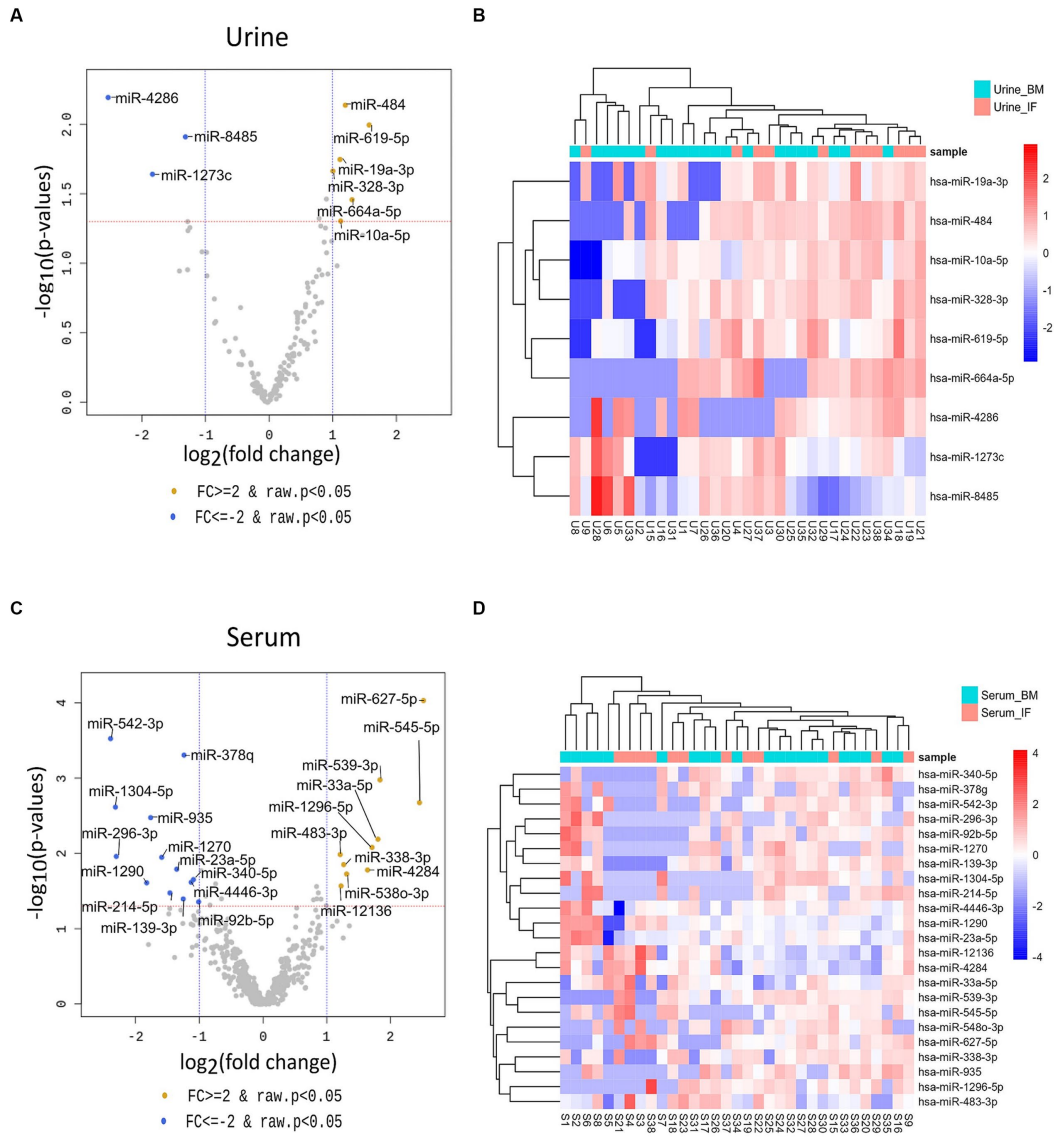
Volcano plots **(A,C)** of differentially expressed urine and serum exosomal miRNAs in the two groups using DESeq2 for data analysis. The log2 fold change (log2 FC) indicates difference of comparison, while −log10 (*p*-value) shows the level of significance of each miRNA between two groups. |Fold change| ≥ 2 and *p*-value < 0.05 were used to identify differentially expressed miRNAs between the two groups. Differences were visualized using R 3.6.1 (www.r-project.org). The heatmap **(B,D)** of DEmiRNAs was constructed using “pheatmap” R package (Kolde, Raivo (2019). Pheatmap: Pretty Heatmaps. R package version 1.0.12. https://CRAN.R-project.org/package=pheatmap).

To identify miRNAs of exosomes derived from urine and serum samples that might be associated with complications in preterm infants, differentially expressed miRNAs (DEmiRNAs) between breastmilk-fed and formula-fed groups were analyzed. A total of 2,656 mature miRNAs with zero counts across more than 51% of all samples were excluded, leaving 154 and 469 mature miRNAs for BM and IF groups, respectively. After statistical test using exactTest and Fold Change, nine and 23 miRNAs had an abundance with small *p*-value (*p* < 0.05) in urine and serum samples, respectively. Results of comparing differential expression levels of miRNAs in urine and serum of BM and IF groups are depicted in Figure 3. Table 2 presents the most predominantly up- and downregulated miRNAs in BM-fed group of urine and serum samples, respectively. The heat map of DEmiRNAs was illustrated using Pheatmap v1.0.12 in Figures 3B,D. Our sequencing data revealed that six miRNAs (hsa-miR-619-5p, hsa-miR-664a-5p, hsa-miR-484, hsa-miR-19a-3p, hsa-miR-10a-5p, hsa-miR-328-3p) were differentially expressed in urinary EVs and ten miRNAs (hsa-miR-627-5p, hsa-miR-545-5p, hsa-miR-539-5p, hsa-miR-33a-5p, hsa-miR-1296-5p, hsa-miR-4284, hsa-miR-548o-3p, hsa-miR-338-3p, hsa-miR-12136, hsa-miR-483-3p) were significantly up-regulated in serum EVs of BM-fed preterm infants (Table 2). In further analysis, we focused on these six and ten upregulated miRNAs in urine and serum EVs of BM-fed preterm infants, respectively.

**Table 2:** Most differentially expressed exosomal miRNAs in urine and serum (breast-fed vs. formula-fed).

Biofluid	miRNA	logFC	logCPM	*p*-value
Urine	hsa-miR-619-5p	2.980822	11.204031	0.01015517
	hsa-miR-664a-5p	2.472770	8.552271	0.03498467
	hsa-miR-484	2.298957	9.091072	0.0073081
	hsa-miR-19a-3p	2.184316	8.971845	0.04986058
	hsa-miR-10a-5p	2.169134	12.274405	0.01795504
	hsa-miR-328-3p	2.007457	9.626131	0.02175376
	hsa-miR-4286	−5.763903	8.438199	0.006421627
	hsa-miR-1273c	−3.556804	10.060786	0.022904048
	hsa-miR-8485	−2.482458	13.708561	0.012333532
Serum	hsa-miR-627-5p	5.717146	6.305259	9.3367E-05
	hsa-miR-545-5p	5.475290	5.706910	0.002119379
	hsa-miR-539-3p	3.564859	6.816660	0.001055754
	hsa-miR-33a-5p	3.487216	6.859864	0.006475274
	hsa-miR-1296-5p	3.269009	4.937124	0.008332218
	hsa-miR-4284	3.113500	9.124149	0.016633993
	hsa-miR-548o-3p	2.480360	5.009451	0.01880367
	hsa-miR-338-3p	2.402597	6.500702	0.014093223
	hsa-miR-12136	2.334674	10.901402	0.027091412
	hsa-miR-483-3p	2.312663	13.216990	0.010354444
	hsa-miR-542-3p	−5.248159	5.573964	0.000299216
	hsa-miR-1304-5p	−4.975675	4.801230	0.002427662
	hsa-miR-296-3p	−4.930382	5.013698	0.010972196
	hsa-miR-1290	−3.543079	10.821049	0.024603236
	hsa-miR-935	−3.394161	5.265165	0.003353104
	hsa-miR-1270	−3.009292	4.379843	0.011267873
	hsa-miR-214-5p	−2.752111	4.452986	0.033535364
	hsa-miR-23a-5p	−2.556573	8.431524	0.016217351
	hsa-miR-378q	−2.381636	5.007624	0.040298643
	hsa-miR-139-3p	−2.361248	5.775051	0.000495777
	hsa-miR-4446-3p	−2.184703	8.113635	0.024205779	hsa-miR-340-5p	−2.132230	5.346058	0.02229045	hsa-miR-92b-5p	−2.014595	4.895494	0.044094996

### 2.5 miRNA-target evaluation and pathway analysis

**Figure 4:**
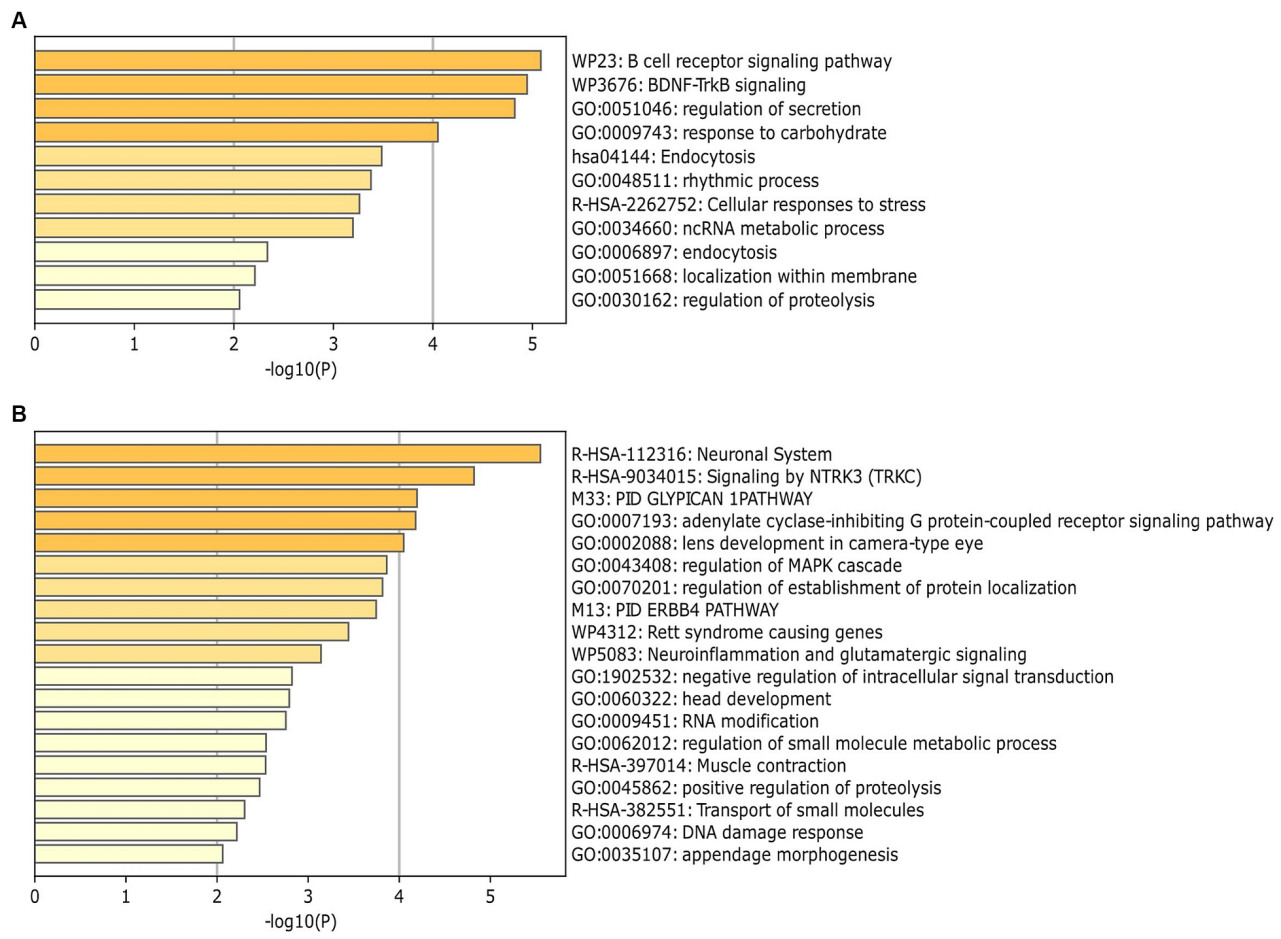
Identification of enrichment ontology (GO and KEGG) terms of miRNA-target genes. **(A)** Top 10 enriched terms of differentially expressed miRNAs (DEmiRNA) associated genes (colored by *p*-values) in urine comparison between BM and IF groups. **(B)** Top 20 enrichment analysis for target of DEmiRNAs in serum comparison between BM and IF groups. Heatmaps of **(A,B)** were produced via Metascape (https://metascape.org).

**Figure 5:**
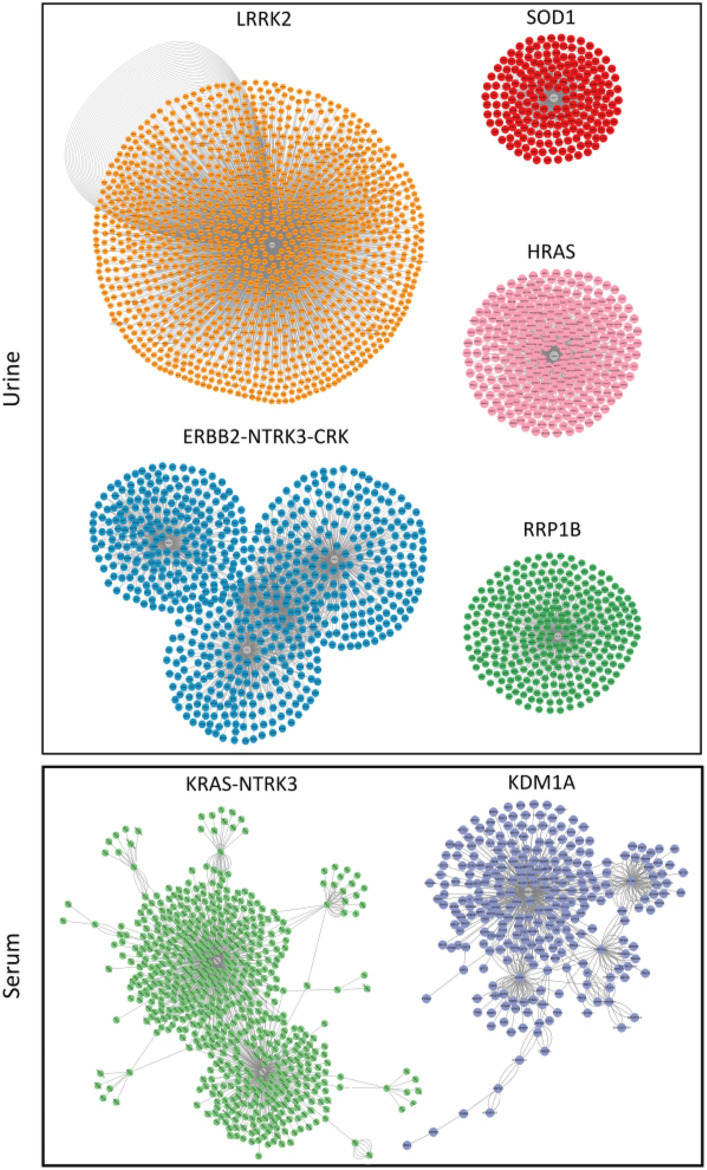
Clusters of proteins generated by clusterMaker 2.0 Cytoscape application with the gLay option. They were derived from protein–protein interaction (PPI) network. Central nodes (gray) represent proteins with higher numbers of interactions. Clusters in urine and serum samples were filtered with a False Discovery Rate (FDR) < 0.05 (Benjaminin Hockberg correction).

MiRNAs listed in Table 2 were exploited as input for MiRWalk analysis, acquiring their validated targets for all binding sites (3′UTR, 5′-UTR and CDS of the target) (Supplementary Table S2). For each biofluid, a distinct target list was established and filtered based on the number of miRNA hits. A threshold of at least five of was set (Supplementary Table S2). These targets were enriched in “B cell receptor signaling pathway” and “BDNF–TrkB signaling” in urine (Figure 4A, Supplementary Table S3). They were enriched in “neuronal system,” “signaling by NTRK3,” and “lens development *in camera*-type eye” in serum (Figure 4B, Supplementary Table S3). In addition, acquired targets were used to create a Protein–Protein Interaction Network (PPI) to demonstrate clusters of proteins retrieved from the IMEx database. A total of 2,877 nodes with 5,814 edges were produced for urine, while serum showed 900 nodes and 1,409 edges. Major protein clusters were selected as criteria with more than 200 interaction thresholds in numerous protein clusters, resulting in five clusters for urine and two clusters for serum (Figure 5, Supplementary Figures S1, S2). These protein clusters represented central nodes, corresponding to target genes having the highest number of interactions with other proteins correlated to similar biological functions. Gene Ontology (GO) enrichment analysis with ClueGO app was performed for these major clusters (Supplementary Tables S4, S5). LRRK2 was one of the central node proteins for urine. GO terms for the LRRK2 cluster related to neuronal function and brain development such as “nervous system development,” and “axon guidance.” Regarding NTRK3-ERBB2-CRK cluster central node, “immune system” and “neurotrophin signaling pathway” were enriched terms. The most enriched GO terms for the HRAS cluster associated with recognizing toxic DNA structures and membrane networking such as “ATP-dependent DNA damage sensor activity” and “membrane trafficking.” “Nervous system development,” “cellular response to stress,” and “neuron death in response to oxidative stress” were enriched GO terms for the cluster with RRP1B central node, RRP1B. Analogous with other central nodes, “peroxidase activity” and “detoxification of reactive oxygen species” were enriched biological processes found for the cluster of SOD1 central nodes. Among the serum cluster, KRAS-NTRK3 was one of the central nodes involved in neural development, namely “axon guidance,” “neuron projection and morphogenesis,” and “neuron differentiation.” Another central node of serum KDM1A seemed to be related epigenetic modification such as “histone modification,” “chromatin organization,” and “regulation of gene expression, epigenetic.” Complete GO term data from ClueGO analysis for all PPI central clusters are listed in Supplementary Tables S4, S5.

## 3 Materials and methods

### 3.1 Ethics approval and informed consent

The present study was approved by the Institutional Review Board (IRB) of Soon Chun Hyang University Hospital Cheon-An (SCHCA 2021-01-029). For all infants participating in this study, their parents or guardians signed a consent form approved by the IRB.

### 3.2 Sample collection

Urine samples (5–25 mL) from 33 infants were collected and kept on ice. They were then transported to a laboratory. Protease inhibitors 1 tablet (cOmpleteTM Protease inhibitor cocktail, Basel, Switzerland) was added to samples immediately to prevent degradation of exosome-associated proteins. Specimens were stored at −80°C. Venous EDTA-stabilized blood samples (*n* = 33) were obtained from infants who were breastfed and infant-formula-fed infants aged 3–4 weeks. Approximately 3 mL of whole blood was collected from each subject. EDTA tubes were clotted at room temperature at least 30 min and centrifuged at 1,300 g for 10 min. Supernatants were collected and transferred to new tube to obtain fresh serum from whole blood after centrifuging at 3,000 g for 20 min at 4°C. These serum samples were kept in a −80°C deep freezer until processed.

### 3.3 Exosomes isolation

Exosomes were isolated from serum samples using ExoQuick (cat. no. EXOQ20A-1; System Biosciences, Palo Alto, CA, United States) to precipitate particles in serum. A high centrifugation method was not suitable to extract exosomes from serum samples because the initial volume of serum was small. In addition, proteins and lipoprotein particles are abundant in the serum Thus, exosomes were isolated from serum samples using ExoQuick according to the manufacturer’s instructions. The pellet was collected and re-suspended in 100 μL of sterilized 1X PBS and then stored at −80°C. Urine samples were centrifuged at 2,000 g for 30 min at 4°C to get rid of cells and debris. Urinary exosomes can be compromised by the presence of huge amounts of Tamm-Horsfall Protein (THP), a urinary protein. This oligomerized protein can trap and sequester exosomes during centrifugation. Remaining THP proteins were removed by further centrifugation at 17,000 g for 60 min at 4°C. The supernatant was saved and the pellet containing THP was incubated with DL-dithiothreitol (DTT, final concentration of 200 mg/mL). DTT reduced disulfide bonds linking the monomers in the THP. During the incubation of DTT and pellet at 37°C for 10 min, samples were well mixed every 2 min. Before transferring supernatant and incubated pellet to clean tubes, they were syringe filtered with 0.80 μm, 0.45 μm, and 0.20 μm filters consecutively for removal of large particles and sterilization. Filtered samples were loaded onto Amicon Ultra-15 Centrifugal Filter Units with Ultracel-30 membrane (MWCO = 30 kDa; Merck Millipore, Billerica, MA, United States) and concentrated to ≤500 μL by centrifugation at 4,000 g repeatedly. The concentrate was transferred to a clean tube for further ultracentrifugation at 110,000 g for 2 h at 4°C using a swinging bucket rotor P40ST (Hitachi, Tokyo, Japan) of himac CP100NX ultracentrifuge. The pellet was collected and re-suspended in 200–300 μL of sterilized 1X PBS and then stored at −80°C until processed

### 3.4 Exosomes characterization

#### 3.4.1 Western blotting

Exosomes derived from all samples were subjected to western blot analyses. Exosome samples were first treated with a RIPA lysis buffer with protease inhibitor. Protein concentration was quantified using the bicinchoninic acid (BCA) method. Absorbance values were detected with a Multiskan GO (Thermo Fisher Scientific, Waltham, MA, United States). Lysates then centrifuged at 14,000 g for 15 min at 4°C. The supernatant was resolved by SDS-PAGE and then immunoblotted with standard techniques. Exosome protein markers against CD9 (1:1000, Abcam, Cambridge, UK) and as a negative control, Calnexin (1:1000, Cell signaling Technology, Danvers, MA, United States), were validated. 293 T cells were used to obtain a cell lysate control compared to exosome markers. Signals of membranes were captured and imaged with a ChemiDocTM Touch Imaging System (Bio-Rad Laboratories, Hercules, CA, United States).

#### 3.4.2 Transmission electron microscopy and nanoparticle tracking analysis

To confirm morphology of exosome particles and determine the presence and purity of exosome, five to ten microliters of exosome from all body fluids were applied to a glow-discharged formvar film on copper 300 mesh grids (catalog no. FCF300-CU) for transmission electron microscopy. Dried grids were washed with distilled water twice and stained for contrast using 2% uranyl acetate 5 μL for 3 s. Samples were examined with a TEM (LIBRA 120, Carl Zeiss, Germany) and images were captured. To confirm concentrations and size distributions of exosome particles, random samples from body fluids were analyzed using a nanoparticle tracking analysis instrument (Nanosight NS300; Malvern Panalytical Ltd., Malvern, UK). Nanoparticle Tracking Analysis is a technique for gaging the size and concentration of nanoparticles in suspension in real time. It is established by tracking the light scattered from suspended particles sustaining Brownian motion. Approximately 10 μL exosomes samples were diluted 1:1,000 to 1:100,000 in sterilized 1X PBS. The dilution was loaded onto a 1 mL syringe and injected into a green laser module. Sizes of particles were analyzed using NTA 3.4 Build 3.4.003 version with an sCMOS camera type. The level of the camera was 15. The number of gains was 250–350 and the temperature was 25°C. The exposure time was automatically placed in the program.

### 3.5 RNA extraction and library preparation

Small size RNAs containing microRNAs were isolated from exosomes samples obtained from body fluids using an miRNeasy Mini kit (Qiagen, Hilden, Germany). Exosome samples were homogenized (IKA Works, Staufen, Germany) with 700 μL QIAzol lysis buffer (Qiagen). Small size RNAs were isolated according to the manufacturer’s instructions. Finally, small size RNAs were precipitated with RNase-free water. Sizes of miRNAs were confirmed using an Agilent RNA 6000 Pico Kit and a Small RNA Kit on Agilent 2100 Bioanalyzer (Agilent Technologies, Santa Clara, CA, United States).

### 3.6 Bioinformatic analysis

#### 3.6.1 From sequencing to dataset

RNAs (10 ng) from each sample were used to construct sequencing libraries with a SMARTer smRNA-Seq Kit (Takara Bio, Kusatsu, Japan) following the manufacturer’s protocol. Validation of libraries was performed using Agilent Technologies 2100 Bioanalyzer by checking the size, purity, and concentration. We estimated the quantity of libraries using qPCR according to qPCR Quantification Protocol Guide (KAPA Library Quantification kits for Illumina Sequencing platforms). These libraries were certified using TapeStation D1000 ScreenTape (Agilent Technologies, Waldbronn, Germany). Libraries were pooled with equimolar amounts and sequenced on an Illumina HiSeq 2500 (Illumina, San Diego, CA, United States) for generating 51 bp reads. Image decomposition and quality values calculation were carried out using modules of the Illumina pipeline. Sequence alignment and detection of known microRNAs were carried out using miRDeep2 software algorithm (Berlin Institute for Medical Systems Biology at the Max-Delbruck-Center for Molecular Medicine, Berlin-Buch, Germany). Prior to executing sequence alignment, *Homo sapiens* hg38 reference genome was searched from UCSC genome browser and indicated using Bowtie (1.1.2^1^) to align sequencing reads to reference sequences. Sequence alignment was then executed for *Homo sapiens* mature and precursor miRNAs obtained from miRBase v21.^2^ All steps of next-generation sequencing analysis were fulfilled with Rokit Genomics (Seoul, Korea).

#### 3.6.2 Differential gene expression analysis

Reads for each miRNA were subjected to Trimmed mean of M-values (TMM) normalization with edge R library (Genome Biology Unit, European Molecular Biology Laboratory, Heidelberg, Germany). For pre-processing, mature miRNAs with zeroed counts across more than 50% of all samples were excluded. We added 1 with normalized read count of filtered miRNAs to facilitate log2 transformation to draw a correlation plot. For each miRNA, logCPM (Counts Per Million) and log fold change were calculated between test and control. A statistical hypothesis test for comparing groups was conducted using exactTest in edgeR. |Fold change| ≥ 2 and *p*-value < 0.05 were used to identify differentially expressed miRNAs between groups. Hierarchical clustering analysis was performed using complete linkage and Euclidean distance to display expression patterns of differentially expressed miRNAs that satisfied the criteria, i.e., |fold change| ≥ 2 and *p*-value < 0.05. Differentially expressed miRNAs were analyzed and visualized using R 3.6.1.^3^

#### 3.6.3 miRNA targets retrieving and functional analysis

To determine functions of the differentially expressed miRNAs in exosomes from biofluids, predicted targets were annotated on miRWalk 3.0,^4^ which was provided data obtained from a machine learning algorithm including experimentally verified miRNA-target interactions. Moreover, functional enrichment analysis was performed using Metascape databases (47) with *p*-value < 0.05 as the cut-off criterion. A protein–protein interaction (PPI) analysis was performed for predicted targets using Cytoscape software v3.9.1 suite (48) and IMEx database (49), which contained nonredundant information derived from the major public protein databases. Afterwards, the clusterMaker 2.0, a Cytoscape application (50) with the “gLay” option, was used to highlight different clusters within the network based on the number and type of connections between nodes. Clusters with several interactions greater than 30 were examined in Gene Ontology (GO) enrichment analysis carried out with ClueGO and CluePedia (51, 52). Results were filtered with an FDR < 0.05 (Benjaminin Hockberg correction).

## 4 Discussion

Evidence is accumulating that exosomes have a potential role in providing us knowledge of pathologic processes and in aiding biomarker discovery for commonly faced conditions in preterm infants (53). Especially, exosomes are easily found in various biological fluids (54–58). They are widely used as research sources by ensuring stability, quality, and quantity of exosomal miRNAs (59, 60). Studies have reported that BM is associated with a reduced incidence of common neonatal complications (19, 21–26). However, the role of miRNAs of exosomes in biological fluids when BM is consumed in preterm infants has not been reported yet. Our first objective was to determine differences in prototypical exosomal miRNAs between BM and IF groups as differences about metabolites in infants fed with BM and IF had been previously documented (17, 61). Our sequencing data revealed that six miRNAs were differentially expressed in urinary exosomes and that 10 miRNAs were significantly up-regulated in serum exosomes of BM-fed preterm infants than in IF-fed infants (Table 2).

A total of 16 upregulated DEmiRNAs in BM were recognized in this study, including miR-619-5p, miR-664a-5p, miR-484, miR-19a-3p, miR-627-5p, miR-33a-5p, and miR-12136. Of them, hsa-miR-619-5p has been reported to be able to bind fully complementary to mRNAs of more than 200 human genes (62), suggesting that it has a broad biological function. MiR-664a-5p has been linked to neuronal differentiation (63) and osteogenic differentiation (64). MiR-484 is located in the Meiosis arrest female1 (MARF1) promoter region on chromosome 16q13.11 in human related to nervous system (65–67). It is increased in miR-484 alleviated cerebral ischemia/reperfusion injury of a mouse model (68). Especially, it has been reported that miR-19a-3p is correlated with sepsis-induced coagulopathy (69) and that it can function as a potential therapeutic target in neonates with sepsis-induced disseminated intravascular coagulation (70). In upregulated miRNAs in the serum, hsa-miR-627-5p can restrain pulmonary artery smooth muscle cell dysfunction by targeting MAP2K4 and PI3K/AKT signaling (71) and chronic lung disease, the most common complication in preterm infants (72). MiR-33a-5p can inhibit viral infection by targeting eukaryotic translation elongation factor 1A1 (*EEF1A1*) (73). Moreover, it has been reported that miR-12136 is highly expressed in the brain tissue and that it plays an crucial role in the development of the nervous system (74). Therefore, these differentially expressed miRNAs in BM (Table 3) can contribute to nervous system development or alleviate neonatal complications, including coagulation, chronic lung disease, and viral infection.

**Table 3:** Target gene consistencies of each biofluids detailed with respect to the binding sites.

Biofluid	miRNA	Target 3′-UTR	Target 5′-UTR	Target CDS	Filtered target
Urine	hsa-miR-619-5p	10,023	1,254	7,650	40
	hsa-miR-664a-5p	4,790	680	4,836	
	hsa-miR-484	6,435	1,447	5,498	
	hsa-miR-19a-3p	488	37	294	
	hsa-miR-10a-5p	2,476	581	3,011	
	hsa-miR-328-3p	6,970	2,026	6,198	
Serum	hsa-miR-627-5p	1,850	244	1,473	87
	hsa-miR-545-5p	64	5	15	
	hsa-miR-539-3p	186	12	102	
	hsa-miR-33a-5p	2,774	319	2,941	
	hsa-miR-1296-5p	7,310	2,069	9,152	
	hsa-miR-4284	5,038	636	3,811	
	hsa-miR-548o-3p	667	85	634	
	hsa-miR-338-3p	2,271	380	4,504	
	hsa-miR-12136	904	73	803	
	hsa-miR-483-3p	7,917	1,782	5,573	

According to gene ontology analysis of DEmiRNAs target genes, ‘B cell receptor signaling pathway’ and ‘BDNF–TrkB signaling’ were the most enriched pathways in urine. These two biological processes mainly contribute to immune responses (75) and protect neurons from cell death caused by reactive oxygen species (76), respectively. Oxygen therapy is frequently used in the treatment of neonatal diseases. However, it can cause neuronal glial cell death and result in neonatal brain injury (77). Consumption of BM is involved in the reduction of metabolites associated with reactive oxygen species in urine of preterm infants than full-term infants (17). In this regard, enrichment analysis results discovered that BM consumption in preterm infants might reduce side effects of oxygen treatment in the NICU. In addition, ‘neuronal system’, ‘signaling by NTRK3’, and ‘lens development *in camera*-type eye’ were enriched GO terms in the serum. A large cohort’s study of preterm infants with 30 weeks of gestation has shown that BM consumption has statistically significant effects on visual acuity (*p* = 0.003) and teller acuity scores rather than IF feeding (*p* < 0.0001) (15). Moreover, a recent study has reported that BM feeding is associated with a reduced odds of stage 2 or 3 ROP (OR = 0.25, 95% CI: 0.091–0.705; *p* = 0.009) in preterm infants (24). That study also claimed that early BM feeding before achieving complete enteral feeding of bovine milk-containing products might alleviate the development of ROP. Taken together, these results suggest that consumption of breast milk may reduce the incidence and severity of neonatal complications.

A total of seven central node genes were recognized by protein network analysis, including LRRK2 (Leucine-Rich Repeat Kinase 2), ERBB2-NTRK3-CRK, HRAS (Harvey rat sarcoma virus), RRP1B (Ribosomal RNA Processing 1B), and SOD1 (superoxide dismutase 1) in urine and KRAS-NTRK3 and KDM1A (Lysine Demethylase 1A) in serum. NTRK3 (Neurotrophic tyrosine kinase, receptor, type 3) was a central node protein for both biofluids. It belongs to the neurotrophic tyrosine kinase family containing c-terminal protein tyrosine kinase domain. It is essential for development and growth of the nervous system (78). Interestingly, NTRK3 is a receptor for neurotrophins. A recent study has reported significant different levels of neurotrophins in preterm infants compared to term infants (79). In line with that study, our study also showed significant differences in levels of miRNAs related to NTRK3 in both urine and serum samples of preterm infants between BM-fed and formula-fed groups. Among central nodes were ERBB2 (v-erb-b2 avian erythroblastic leukemia viral oncogene homolog 2), CRK (Cysteine-rich receptor-like-kinase), HRAS, and KRAS (Kirsten rat sarcoma virus), all of which were proto-oncogenes involved in the development of various cancer types. Their overexpression is also closely related to various diseases (80–82). Data of this study informed that miR-627-5p, miR-4284, miR-33a-5p, miR-1296-5p, and miR-12136 were involved in KRAS protein regulation (Supplementary Table S2). KRAS protein was implicated in congenital pulmonary disease in a recent study (83), suggesting that these DEmiRNAs might be related to neonatal pulmonary disease. Among them, miR-1296 has been suggested as potential biomarker in chronic lung disease in adults (84). Other miRNAs have not been reported in relation to pulmonary disease. These findings suggest that miRNA-627-5p, miR-4284, miR-33a-5p, miR-1296-5p, and miR-12136 require further investigation in cases of neonatal pulmonary disease before they can be used as biomarkers or therapeutic targets. Another function derived from the PPI cluster analysis was immune involvement of RRP1B, also known as nucleolar protein. When RRP1B is overexpressed, it can promote RNA-dependent RNA polymerase binding to capped mRNA and enhance viral transcription of influenza A viruses (85). In addition, it is contagious (86). Moreover, LRRK2 protein, another central cluster protein supported by the network analysis (Figure 5), is concerned with lysosomal activity in neurons with a role in the autophagy pathway (87). Interestingly, a recent study has shown that autophagy is dependent on oxidative stress and that inhibiting autophagy can significantly reduce ischemic damage in a neonatal rat brain model (88). SOD1 is an enzyme constituting important antioxidant defense against oxidative stress in human body that has therapeutic effects on various disease (89). Some studies on prematurity have suggested that haplotypes of SOD1 genes might be independent protective or risk indicators for complications of prematurity (90). Poggi et al. have reported that SOD1 can reduce the risk of RDS, IVH, and ROP in a study of 152 preterm infants (91). Regarding the involvement of other antioxidant genes, KDM1A that can regulate homeostasis of histone methylation (92) has been reported to play a role in regulating autophagy. It is involved in the transcriptional control of various downstream effectors (93). Especially, LSD1 participates in antioxidant gene expression with NRF2 (nuclear factor erythroid 2-related factor 2) (94). It may reduce oxidative stress. Accumulating evidence of various effects of BM on preterm infants related to immune response, nervous system, eye development, and brain development could improve neonatal conditions in NICUs. Despite advances in NICUs, preterm infants often suffer from neurological disorders in later life. Premature infants are especially vulnerable to injury caused by ROS due to their imperfect endogenous radical scavenging systems (77). In this regard, exploiting involvement in antioxidant processes through analysis of DEmiRNAs and their target genes in BM could lead to useful therapies for preterm infants with specific prevention strategies against several complications.

In summary, we found that intake of BM and IF in NICU caused practical differences in exosomal miRNA contents in urine and serum samples of preterm infants. Increased miRNAs and their targets were relevant for neural development, ROS regulation, and detoxification processes. It would be interesting to improve neonatal status and further develop neuronal system containing brain and eye. While this is intriguing, the impact of certain amounts of transcription on gene expression needs to be evaluated. The amount of efficacy on cells or tissues of preterm infants needs to be assessed in all aspects to elucidate their roles through *in vitro* and *in vivo* experiments.

## Data availability statement

The original contributions presented in the study are publicly available. The raw sequence datasets generated for this study can be found in SRA. BioProject ID collecting all samples is the following: PRJNA1035999.

## Ethics statement

The studies involving humans were approved by Investigational Review Board (IRB) of Soon Chun Hyang University Hospital Cheon-An (SCHCA 2021-01-029). The studies were conducted in accordance with the local legislation and institutional requirements. Written informed consent for participation in this study was provided by the participants’ legal guardians/next of kin.

## Author contributions

E-BK: Conceptualization, Data curation, Formal analysis, Visualization, Writing – original draft. JS: Investigation, Validation, Writing – review & editing. LL: Investigation, Data curation, Writing-review & editing. HK: Data curation, Methodology, Software, Writing – original draft. JK: Investigation, Methodology, Software, Writing – original draft. YS: Methodology, Software, Validation, Writing – original draft. HJ: Data curation, Software, Visualization, Writing – original draft. H-TK: Project administration, Supervision, Writing – review & editing. SR: Conceptualization, Project administration, Supervision, Validation, Writing – review & editing.

## Footnotes

^1^ http://bowtie-bio.sourceforge.net/

^2^ http://www.mirbase.org/

^3^ http://www.r-project.org

^4^ http://mirwalk.umm.uni-heidelberg.de/

## Supplementary material

SUPPLEMENTARY FIGURE S1 Clusters of proteins generated by clusterMaker 2.0 Cytoscape application using differential expressed miRNAs targets in urine. miRNA Targets were filtered based on established criteria.

SUPPLEMENTARY FIGURE S2 Clusters of proteins generated by clusterMaker 2.0 Cytoscape application using differential expressed miRNAs targets in serum. miRNA Targets were filtered based on established criteria.

SUPPLEMENTARY TABLE S1 Mapping statistics by smallRNA library sequencing.

SUPPLEMENTARY TABLE S2 List of genes selected by targeting 5 or more miRNAs in each sample, and miRNA’s target gene consistency for binding sites.

SUPPLEMENTARY TABLE S3 Functional enrichment analysis results from Metascape with the target gene list selected in S2.

SUPPLEMENTARY TABLE S4 Gene Ontology enrichment results of clstuer analysis in Urine.

SUPPLEMENTARY TABLE S5 Gene Ontology enrichment results of clstuer analysis in Serum.
