# Supplementary figures and images for: Characterization of exosomal microRNAs in preterm infants fed with breast milk and infant formula
Source: Front Nutr. 2024 Jan 18;11:1339919. doi: 10.3389/fnut.2024.1339919 (PMC10830786; doi:10.3389/fnut.2024.1339919)

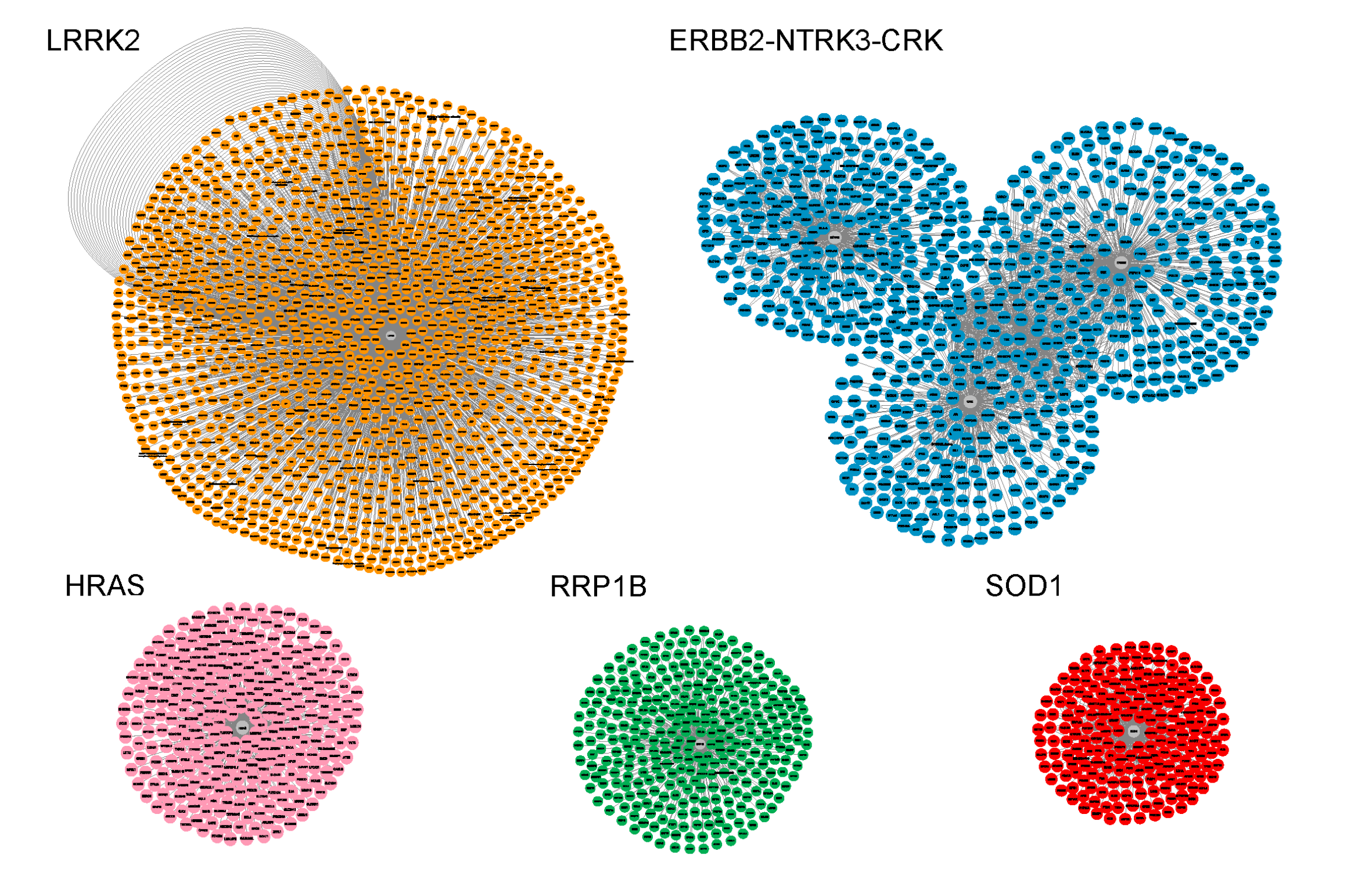

Supplement: SUPPLEMENTARY FIGURE S1 — Clusters of proteins generated by clusterMaker 2.0 Cytoscape application using differential expressed miRNAs targets in urine. miRNA Targets were filtered based on established criteria. [file Image_1.PNG]

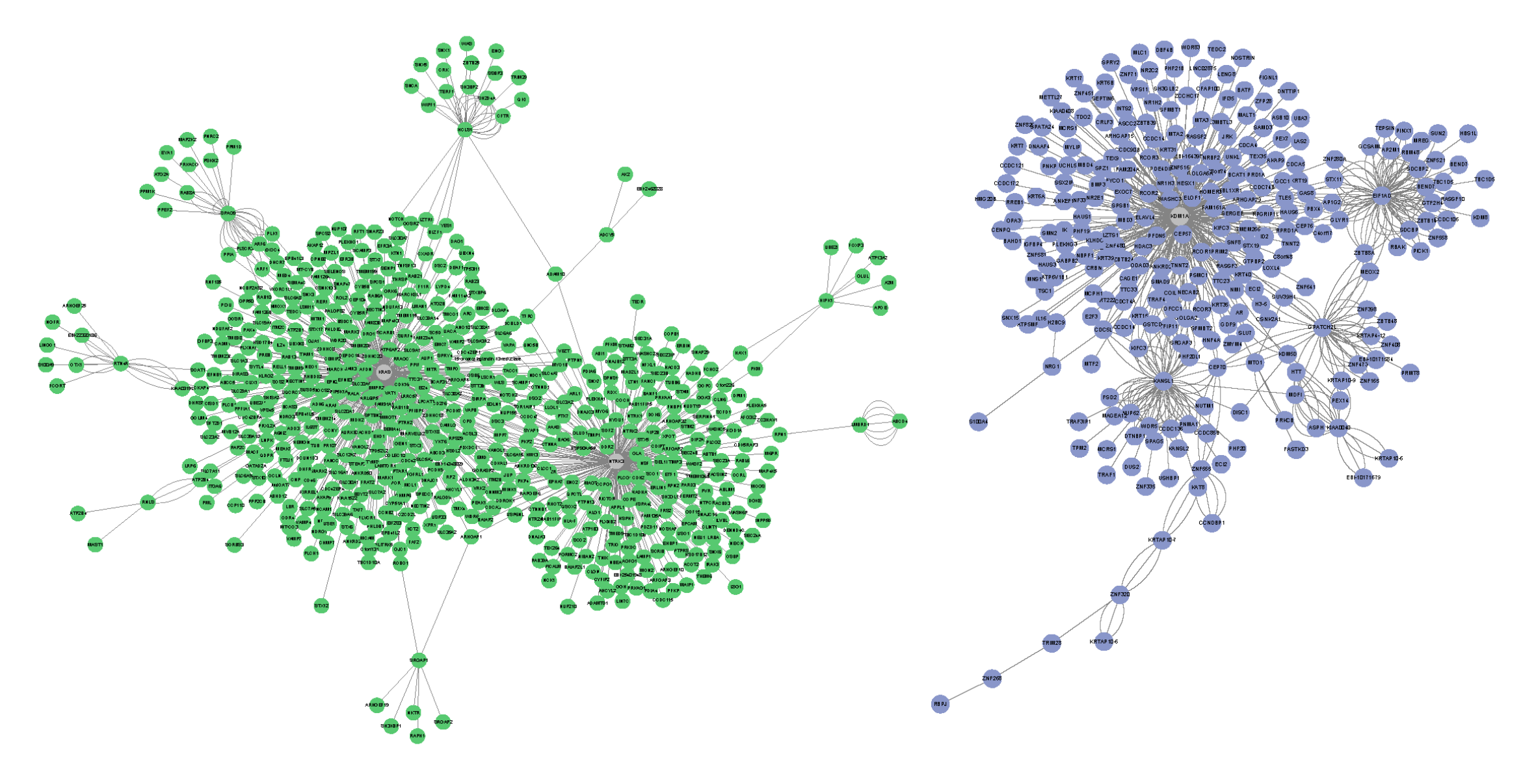

Supplement: SUPPLEMENTARY FIGURE S2 — Clusters of proteins generated by clusterMaker 2.0 Cytoscape application using differential expressed miRNAs targets in serum. miRNA Targets were filtered based on established criteria. [file Image_2.PNG]
